# Population Genetic Structure and Hybridization of *Schistosoma haematobium* in Nigeria

**DOI:** 10.3390/pathogens11040425

**Published:** 2022-03-31

**Authors:** Amos Mathias Onyekwere, Olivier Rey, Jean-François Allienne, Monday Chukwu Nwanchor, Moses Alo, Clementina Uwa, Jerome Boissier

**Affiliations:** 1 Department of Biology, Alex Ekwueme Federal University, Ndufu-Alike, Abakaliki PMB 1010, Nigeria; onyekwereamos@gmail.com (A.M.O.); uwaamakamma@gmail.com (C.U.); 2 IHPE, University Montpellier, CNRS, Ifremer, University Perpignan Via Domitia, F-66000 Perpignan, France; olivier.rey@univ-perp.fr (O.R.); allienne@univ-perp.fr (J.-F.A.); 3 Department of Zoology, Nnamdi Azikiwe University, Akwa PMB 5025, Nigeria; mnwanchor@gmail.com; 4 Department of Microbiology, Alex Ekwueme Federal University, Ndufu-Alike, Abakaliki PMB 1010, Nigeria; gianimarg@gmail.com

**Keywords:** Nigeria, hybrids, population genetic analysis, *Schistosoma* *haematobium*, *Schistosoma* *bovis*

## Abstract

Background: Schistosomiasis is a major poverty-related disease caused by dioecious parasitic flatworms of the genus *Schistosoma* with a health impact on both humans and animals. Hybrids of human urogenital schistosome and bovine intestinal schistosome have been reported in humans in several of Nigeria’s neighboring West African countries. No empirical studies have been carried out on the genomic diversity of *Schistosoma* *haematobium* in Nigeria. Here, we present novel data on the presence and prevalence of hybrids and the population genetic structure of *S*. *haematobium*. Methods: 165 *Schistosoma*-positive urine samples were obtained from 12 sampling sites in Nigeria. *Schistosoma* *haematobium* eggs from each sample were hatched and each individual miracidium was picked and preserved in Whatman^®^ FTA cards for genomic analysis. Approximately 1364 parasites were molecularly characterized by rapid diagnostic multiplex polymerase chain reaction (RD-PCR) for mitochondrial DNA gene (Cox1 mtDNA) and a subset of 1136 miracidia were genotyped using a panel of 18 microsatellite markers. Results: No significant difference was observed in the population genetic diversity (*p* > 0.05), though a significant difference was observed in the allelic richness of the sites except sites 7, 8, and 9 (*p* < 0.05). Moreover, we observed two clusters of populations: west (populations 1–4) and east (populations 7–12). Of the 1364 miracidia genotyped, 1212 (89%) showed an *S*. *bovis* Cox1 profile and 152 (11%) showed an *S*. *haematobium* cox1 profile. All parasites showed an *S*. *bovis* Cox1 profile except for some at sites 3 and 4. *Schistosoma* miracidia full genotyping showed 59.3% of the *S. bovis* ITS2 allele. Conclusions: This study provides novel insight into hybridization and population genetic structure of *S*. *haematobium* in Nigeria. Our findings suggest that *S*. *haematobium* x *S*. *bovis* hybrids are common in Nigeria. More genomic studies on both human- and animal-infecting parasites are needed to ascertain the role of animals in schistosome transmission.

## 1. Introduction

Schistosomiasis is one of the major neglected tropical diseases with public and veterinary health concerns and is endemic in tropical and subtropical regions. With a global burden of about 1.4 million disability-adjusted life years (DALYs), the disease is ranked second after malaria based on morbidity [1]. The *Schistosoma* genus shows a wide definitive host spectrum that ranges from humans to domestic and wild animals. Humans could be infected with one or more of the six human-infecting Schistosome species, and this may lead to combined disease symptoms and co-morbidities. Four human-infecting parasites (*Schistosoma haematobium*, *S. mansoni*, *S. intercalatum*, and *S. guineensis*) are common in Africa, while two (*S*. *japonicum* and *S*. *mekongi*) are prevalent in Asia [2]. Except for *S. intercalatum*, all schistosome species are known to infect domestic or wild mammalian animal hosts [3]. The disease is less recognized in the veterinary sector including livestock and wild animals [4].

In Africa, three species of schistosomes are concerned with livestock infection: *S*. *bovis* and *S. mattheei* infect members of the orders of Cetartiodactyla (mainly Bovidae), Rodentia, Primates, and Perissodactyla and *S. curassoni* infect members of the family of Bovidae [3]. There is little or no documentation available on the prevalence, intensity, repartition, transmission dynamics, and phylogeography of these livestock schistosomes [5,6]. In addition to their veterinarian importance, the animal infecting schistosomes have recently received peculiar interest due to their potential zoonotic importance through hybridization with human-infecting parasites. In Africa, hybrids between *S. haematobium* and the three livestock schistosomes have been evidenced in humans: *S. haematobium* x *S. bovis* [7,8,9], *S. haematobium* x *S. mattheei* [10,11], and *S. haematobium* x *S. curassoni* [12].

The hybrid from human uro-genital schistosome (*S*. *haematobium*) and bovine intestinal schistosome (*S*. *bovis*) is certainly the most studied hybrid form. *S. haematobium* x *S. bovis* hybrids have been reported in infected humans in several West African countries like Benin, Cote d’Ivoire, Mali, Niger, and Senegal [4,9]. No empirical studies have been carried out in Nigeria to identify the presence of hybrids as has been conducted in some of its neighboring countries. Hybrids are commonly identified based on a single nuclear and single mitochondrial marker. A discordance in species assignation on these two markers or the presence of animal parasite allele or haplotype in humans is thus generally considered a hybrid parasite. Based on such an approach, studies have shown hybridization between: *S*. *haematobium* and *S*. *mansoni* [13,14,15], *S*. *haematobium* and *S. bovis,* [7,9,12,16,17,18,19,20,21], *S*. *haematobium* and *S. guineensis* [22,23,24,25], *S. haematobium* and *S. curassoni* [12], or *S*. *haematobium* and *S. mattheei* [10,11,26].

If the presence/absence of *S. haematobium* hybrids have been assessed in some west African countries, the population genetic structure and diversity of this parasite have received less attention. Nevertheless, genomic introgression through hybridization is expected to influence both the genetic diversity and the population structure. Most population genetic studies have been conducted on *S. mansoni* and very few on *S. haematobium*. Some common features can be observed despite the *Schistosoma* species: such as few barriers to gene flow at a local scale or the most important genetic variation within than between definitive hosts [27]. However, *S. haematobium* is peculiar from other species because it presents less genetic diversity and less genetic structure compared to *S. mansoni* or *S. bovis* [27]. To date, a single study has analyzed both the hybrid status of the parasite using the nuclear/mitochondrial marker discordance approach and population genetic structure based on microsatellite markers [28]. This last study showed no clustering when parasites are grouped according to their hybrid versus pure status.

Our study aims to complete our knowledge of *S. haematobium* in the identification of the presence and prevalence of *S. haematobium* versus *S. bovis* hybrids in Nigeria on a large geographical scale as well as microsatellites genotyping to analyze the population genetic structure and diversity.

## 2. Results

### 2.1. Schistosome Genotyping Using Cox1 and ITS2

We obtained a total of 4007 miracidia from 165 *Schistosoma*-positive urine samples and analyzed 1364 successful miracidia. Of the 1364 miracidia, 1212 (89%) and 152(11%) showed an *S. bovis* Cox1 profile and *S. haematobium* Cox1 profile, respectively (Table 1). All studied sites except sites 3 and 4 showed 100% of Cox1 haplotypes. No miracidium was identified as an *S. mansoni* Cox1 profile. Full genotyping (i.e., Cox1 and ITS2 sequencing) was obtained from a sub-sample. The *Schistosoma* miracidia full genotyping showed 6 (5.1%) for the nuclear gene of *S*. *haematobium* (*Sh* x *ShSh*), 46 (39.0%) alleles were assigned to *S*. *bovis* (*Sb* x *SbSb*), and 66 (55.9%) alleles were assigned to *S*. *bovis* x *S*. *haematobium* hybrids (*Sb* x *ShSh*, *Sb* x *SbSh*, and *Sh* x *ShSb*), while no hybrid parasite was identified as *Sh* x *SbSb* genotype (Table 2).

### 2.2. Cox1 Phylogenetic Trees

On the 59 Cox1 sequences we have sequenced 21 different haplotypes: 2 *S. haematobium* and 19 *S. bovis* haplotypes. An *S. bovis* Cox1 phylogenetic tree performed with the haplotypes recovered from Nigeria and several other haplotypes from Cameroon, Benin, Senegal, Ivory Coast, Kenya, and Tanzania did not reveal any spatial structuration (see Supplementary Figure S2).

### 2.3. Microsatellite Analysis

No significant deviation from HW equilibrium or linkage disequilibrium was observed across loci. The genetic variability indices (He, A, Ar, and Fis) are shown in Table 3 from the 14 microsatellite loci. Mean heterozygosity across the population ranged from 0.527–0.598 but we did not observe any significant difference (*p* > 0.05). For allelic richness (Ar), the mean values ranged from 4.718–6.929. Significant differences were observed between site 9 and sites 2 to 4 (*p* < 0.05).

### 2.4. Population Genetic Structure

**Figure 1:**
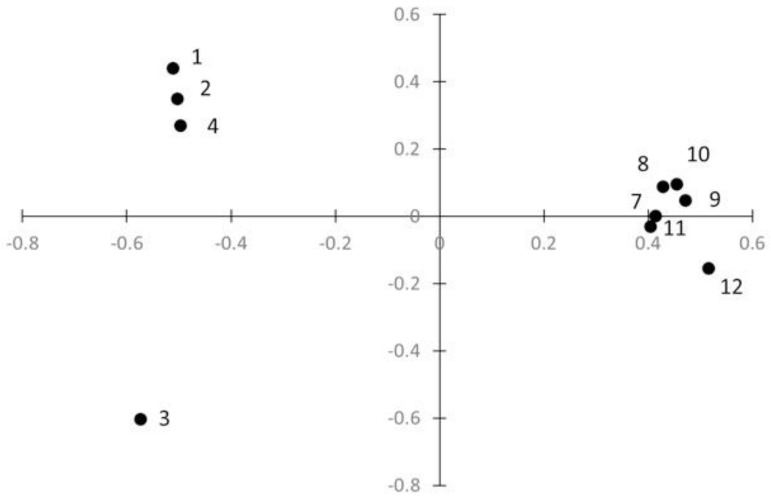
Population genetic structure graph assessed by principal component analysis of 1136 *S. haematobium* parasites collected in Nigeria revealed by graph (PCA). Each sampling site is represented by a dot. The first and second axis of the PCA represent 43.8% and 22.9%, respectively, of the total variation in allele frequency.

**Figure 2:**
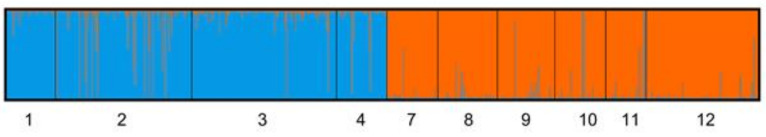
Bar plot showing the population genetic structure using Structure software of 1136 *S*. *haematobium* miracidia collected in Nigeria. Each column represents one miracidium. The colors show the proportion of contribution of each cluster to each genotype. The cluster structure K = 2, produced by structure software for 10 sampling sites.

The pairwise genetic differentiation estimates (F_ST_) between most of the sampling sites are statistically significant after Bonferroni correction, except for between sites 1 and 2, 8 and 9, 8 and 10, 9 and 10, 7 and 11, and 7 and 12 (Table 4). The PCA (Figure 1) showed a structuration among populations with the population from the west (1–4) separated from the population in the east (sites 7–12). Random sampling of two miracidia by patient does not change the latter result (Supplementary Figure S3) This genetic structure was confirmed using Structure software that revealed the highest probability for two clusters (K = 2) (Figure 2).

## 3. Discussion

We report for the first time the population genetic structure and hybridization of *S*. *haematobium* in Nigeria. Based on the Cox1 profile, our study revealed a country-wide minimum proportion of 89% prevalence of *S*. *bovis* x *S*. *haematobium* hybrids and almost equal repartition among the study sites. Most studied sites revealed a hybrid prevalence of 100% except for sites 3 and 4. *S*. *bovis* x *S*. *haematobium* hybrid prevalence obtained from other West African countries: Cote d’Ivoire 57.5% [9] and Senegal 9–72% [7,8,12,28,29,30] revealed that Nigeria has the highest prevalence of hybrids. An important variation in hybrid frequency, ranging from 2% to 26%, between different villages has been evidenced in Senegal [29]. The authors have positively associated this variation with the prevalence of *S. mansoni*. They hypothesized that a first schistosome infection would favor ongoing infections and subsequently hybridizations. Because hybrid prevalence is 100% in the majority of the sites we have sampled, we cannot test for an eventual link with proximal factors such as prevalence and socio-demographic factors we have measured [31].

We have obtained full genotypes (both Cox1 and ITS2) for a sub-sample of 59 parasites. Interestingly, we found a high percentage (39%) of *Sb* x *SbSb* genotypes. This genotype has not been found in Senegal [8,12,28,29] and is in a very low percentage in Cote d’Ivoire [32] and in Corsica [18]. The high percentage of *Sb* x *SbSb* genotype we found is associated with a preponderance of both *S. bovis* Cox1 haplotype and *S. bovis* ITS2 alleles compared to *S. haematobium*. Concerning the ITS2, in Cote d’Ivoire and in Senegal, the frequency of the *S. haematobium* allele is 87% and more than 88%, respectively [8,12,28,29]. We found only 40% of *S. haematobium* ITS2 alleles in Nigeria. When a population is at equilibrium, the ITS is expected to harbor a single allele from one of the parents resulting in a concerted evolution [33]. This supposes that in Nigeria, contrary to other countries the population of hybrid schistosomes is not stabilized.

Concerning Cox 1, in Cote d’Ivoire and Senegal, the frequency of the *S. haematobium* haplotype is 46% and more than 77%, respectively [8,12,28,29]. We found only 11% of the *S. haematobium* haplotype in Nigeria, and these haplotypes were restricted to two sites. As previously proposed by Boon et al. [29], two main factors could explain a variation in Cox1 haplotype frequency: genetic drift and/or selection. Because the mitochondrial genes are only maternally inherited, they are more prone to genetic drift compared to bi-parentally (i.e., nuclear) inherited markers. Mate choice or mate competition could select for a given mitochondria species. Recently, this has been shown in random mating between *S. haematobium* and *S. bovis* excluding the selection of mitochondria through sexual selection [34]. Boon et al. [29] proposed that the environment could select for different mitochondrial haplotypes. For instance, these authors hypothesized that the snail strain host could select for hybrid parasites in a given area. This interesting snail driver selection hypothesis needs to be tested.

Concerning Nigeria, the high frequency of *S. bovis* genes could be explained by active zoonotic transmission and ongoing gene flow between animal (i.e., *S. bovis*) and human parasites. Recent genomic studies have shown that the *S. haematobium* x *S. bovis* hybrid is certainly the result of an ancient introgression event [35,36]. The age of the hybridization does not exclude ongoing zoonotic transmission. This zoonotic transmission has been evidenced in Benin with cows and rodents [20,21] and only with rodents in Senegal [19]. *S. haematobium* x *S. bovis* hybrids have not been evidenced in cows in Cameroon [6]. Considering the high prevalence of *S. bovis* genes in parasite-infecting humans in Nigeria, looking for the presence of hybrid schistosomes in animals (rodents or cows) seems necessary.

To determine the genetic structure among the populations, we measured the pairwise genetic estimates (F_ST_ values), for all pairs. Generally, values < 0.05, 0.05–0.15, 0.15–0.25 and >0.25 indicate low, moderate, high and very high genetic differentiations respectively [6]. Our study revealed F_ST_ values of 0.0104–0.1688 which is an indication of low to very high genetic differentiation among the populations. Few population genetic studies involved *S. haematobium* compared with *S. mansoni* [36], and the studies involved local scales, between 8 and 45 km distances between sites for Gower et al. [37] and Boon et al. [28], respectively. Our study proposed a wider range from local (10’s of kilometers) to regional (10s to 100s of kilometers) scale. When populations are separated by a few kilometers, paired F_ST_ values are in agreement with previous studies and range from 0.01 to 0.04 [37]. At the regional scale, the Fst values for *S. haematobium* are similar to *S. mansoni* [38,39].

Regardless of the method used (PCA or Bayesian analyses using Structure software), we showed a clear clustering into two groups of populations: one from the west (populations 1–4) and one from the east (populations 7–12). *S. haematobium* populations are usually not well structured compared to *S. mansoni* [36]. These two parasite species possess similar transmission dynamics that could influence the parasite’s genetic structure. For instance, for both species, the transmission is focused on water bodies, the intermediate and definitive hosts have similar mobility, and the number of intermediate host species is restricted. It is well established that *S. haematobium* has less genetic diversity than *S. bovis* or *S. mansoni* [6,36]. This low genetic diversity reduces the power of determination of structuring units. Our study shows that no structuring units are detectable under around 250 km distances between populations. Fst values are also lower among populations 1–4 or among populations 5–10 than between the two clusters of populations. In comparison, it has been shown that clear population structures between *S. mansoni* populations are separated by a 127 km distance in Ethiopia [39].

Various factors including hybridization can favor genetic structure [27]. Introgression through hybridization can influence the genetic structure by adding new alleles in a given area and in turn favor population clustering. We have obtained 100% of hybrids in the majority of sites we have sampled. Nowadays, this does not exclude the influence of hybridization in genetic structuring. Indeed, the molecular barcoding method we have used only infer the presence/absence of hybrids and not the genomic introgression level. Molecular markers such as SNP are needed to infer the role of hybridization in genetic structuring.

## 4. Materials and Methods

### 4.1. Parasitological Survey and Sampling Collection

#### 4.1.1. Study Area and Study Population

**Figure 3:**
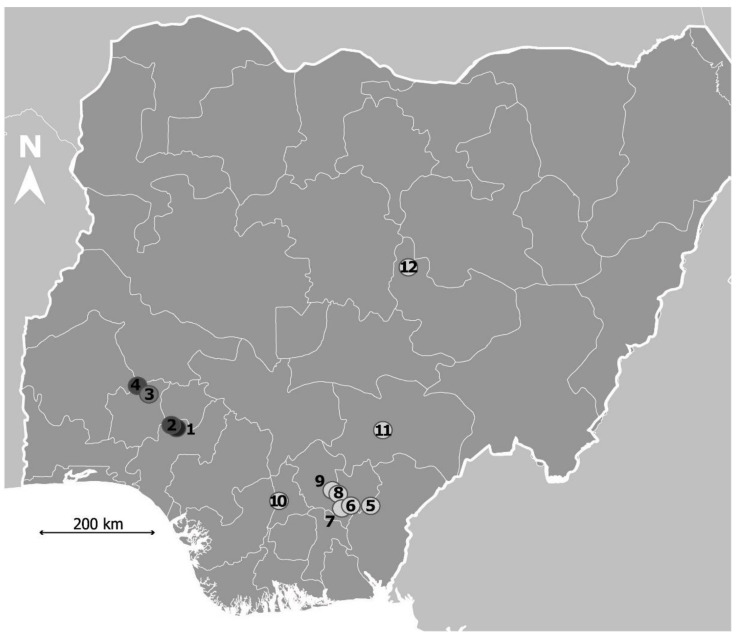
Map showing sampling sites 1–12 in the survey study carried out to determine the prevalence of *S*. *haematobium* infection among primary school-age pupils across Nigeria (Onyekwere, et al. Submitted). Sampling sites were represented according to infection status of the disease among the participants. Darker to lighter colors correspond to higher to lower infection status observed at the studied sites.

This study was carried out in twelve sites in Nigeria, West Africa (Figure 3). This study was integrated into a survey carried out on prevalence and risk factors associated with urinary schistosomiasis among primary school-age pupils in Nigeria (Onyekwere et al. submitted).

#### 4.1.2. Urine Sample Collection and Miracidia Sampling

A labeled, clean, and sterile plastic container with an “identification code” for anonymity of 20 mL was given to each patient whose parents or legal guardians gave oral consent. Each participant whose urine sample was positive for the parasite was treated with a single oral dose of 40 mg/kg body weight of praziquantel (600 mg, Biltricide, Bayer, Leverkusen, Germany) through their Primary Health Center (PHC).

Individual miracidium was harvested using a P10 Gilson micropipette in 3 μL of water under a 20× or 40× magnification binocular microscope. About 20–25 miracidia were individually captured for each participant with each miracidium being checked in the pipette tip before placing on Whatman FTA^®^ cards (GE Healthcare Life Sciences; Amersham, UK). Each FTA^®^ card filled with miracidia was stored at room temperature while on the field and transferred to “Laboratoire Interactions Hotes-Pathogenes-Environnements” (IHPE), France, for genetic analysis. Table 5 shows the number of miracidia collected from participants and genotyped with Cox1 and microsatellites for each of the sampling site.

### 4.2. Genomic Analysis

#### 4.2.1. DNA Extraction

Genomic DNA from *Schistosoma* randomly selected miracidia were individually extracted from FTA^®^ cards using the Chelex method [40]. Harris-Micro-Punch (VWR; London, UK) was used to perforate a 2 mm disc at the center where the sample was placed. The disc was washed in 50 μL ultra-pure water for 10 min, the water discarded, and the disc was incubated in 80 μL of 5% Chelex^®^ solution (Bio-Rad; Hercules, CA, USA) at 65 °C for 30 min with agitation. This was incubated again at 99 °C for 8 min without agitation. The solution was centrifuged at 14,000 rpm for 2 min and 60 μL of the supernatant was transferred into a 96-wells micro-plate and stored at −20 °C for genomic analysis.

#### 4.2.2. Estimation of Hybrid Prevalence by Mitochondrial DNA Identification

Hybrid schistosomes are generally characterized by the combination of the maternal DNA (mt-DNA) from Cox1 and the nuclear DNA (rDNA) from ITS2 [7,9]. The results will be used to assign each parasite a genetic signature based on the haplotype-alleles combinations: *Sb* x *SbSb*, *Sb* x *ShSh*, *Sb* x *SbSh*, *Sh* x *ShSh*, *Sh* x *SbSb* or *Sh* x *SbSh*. We obtained this full genotyping only for a subsample of miracidia (see below). However, a basic estimation of the hybrid frequency could be assessed only by the Cox1 gene characterization considering that human infected by an animal parasite (i.e *S. bovis*) gene is a hybrid parasite [29]. Hence, the frequency of hybrids is a synonym for the frequency of miracidia with an *S. bovis* Cox1 profile. This method can lead to the underestimation of the frequency of hybrids because *Sh* x *SbSb* or *Sh* x *SbSh* genotypes are considered as pure *S. haematobium* instead of hybrids. For this purpose, each miracidium was molecularly characterized by rapid diagnostic multiplex PCR (RD-PCR) on Cox1 [41]. The number of miracidia tested per site is presented in Table 1. We used species-specific primers to amplify the region to discriminate each *Schistosoma* species fragment: *S*. *haematobium* 120 bp, *S*. *mansoni* 215 bp, and *S*. *bovis* 260 bp [41]. The primers we used were a single universal reverse primer; (Shmb.R, 5-CAA GTA TCA TGA AAY ART ATR TCT AA-3′) and three species-specific forward primers; (Sh.F, 5′-GGT CTC GTG TAT GAG ATC CTA TAG TTT G-3′) for *S. haematobium*, (Sm.F, 5′-CTT TGA TTC GTT AAC TGG AGT G-3′) for *S. mansoni* and (Sb.F, 5′-GTT TAG GTA GTG TAG TTT GGG CTC AC-3′) for *S. bovis*. Each PCR is made up of 1.2 μL of ultra-pure water, 2 μL of buffer (Green GoTaq Flexi buffer, 5×; Promega; Madison, Wisconsin, USA), 1.2 μL of 25 mM MgCl2 (Promega), 0.4 μL of 10 mM dNTPs mix (Promega), 1 μL of 10X primer mix (4 μL of 100 μM reverse primer, 4 μL of each 100 μM forward primer and 84 μL of ultra-pure water), 0.2 μL of 5 U/ μL of GoTaq G2 Hot Start Polymerase (Promega), and 4 μL of DNA extract, making a total volume of 10 μL for the PCR mix. Thermal cycling was performed in (plate thermal cycler) a PerkinElmer 9600 Thermal Cycler (PerkinElmer, Waltham, MA, USA) and the PCR conditions used were: pre-denaturing at 95 °C for 3 min; 45 cycles of 10 s at 95 °C (denaturing), 30 s at 52 °C (annealing), and 10 s at 72 °C (extending). This was followed by a final extending period of 2 min at 72 °C. The PCR product was stored in the refrigerator at 4 °C until use. The PCR products (Cox1) were visualized on 2% agarose gel stained with 8 μL Midori dye. Nine microliters of the PCR product was loaded into each well using a multi-channel micro-pipette (including wells for positive controls; *S. haematobium, S. mansoni, S. bovis*, and water for a negative control) and 4 μL for size standard 100 bp (base-pair) ladder. The PCR products in the gel were analyzed by electrophoresis at 135 V for 30–35 min and transferred to the UV trans-illuminator where gel images were taken.

#### 4.2.3. Mitochondrial DNA (Cox1) and Nuclear Internal Transcribed Spacer II (ITS2) Sequencing

Full genotyping on a sub-sample was assessed by SANGER sequencing of the two genes (Cox1 and ITS2). Cox1 and ITS2 genes of six to seven miracidia harboring *S. bovis* Cox1 RD-PCR profile were sequenced for all sites. Seven more miracidia were sequenced for sites 3 and 4, the only sites harboring the *S. haematobium* Cox1 RD-PCR profile (see results). *S*. *haematobium* Cox1 PCR mix was performed in 96 wells with a single forward primer COI1_F: 5′-GGGGGTTTTATTGGTTTAGGTT-3′ and a single reverse primer COI1_R: 5′-CCAATTATAAAAGGCCATCACC-3′, while *S*. *bovis* COI1 PCR mix was performed in 96 wells with a single forward primer COI1_F: 5′-GAGGTGGTTTTATTGGTCTTGG-3′ and a single reverse primer COI1_R: 5′-GGCCACCATCATACCAACAT. Schistosome ITS2 PCR mix was performed with a single forward primer ITS4_F: 5′-TAACAAGGTTTCCGTAGGTGAA-3′ and a single reverse primer ITS5_R: 5′-TGCTTAAGTTCAGCGGGT-3′ (Kane and Rollinson, 1994). The PCR mix was made up of 17.35 μL of ultra-pure water, 6 μL of buffer (colorless GoTag Flexi buffer 5X; Promega; Madison, Wisconsin, USA) 1.8 μL of 25 mM MgCl2 (Promega), 0.6 μL of 10 mM dNTPs mix (Promega), 1 μL of each 10μM primer, 0.25 μL GoTaq G2 Hot Start polymerase (Promega), and 2 μL of DNA extract, making a total volume of 30 μL for each PCR mix. The PCR thermal cycling conditions used was the same for all markers and was performed in (plate thermal cycler) a PerkinElmer 9600 Thermal Cycler (PerkinElmer, Waltham, MA, USA): pre-denaturing at 95 °C for 3 min, 45 cycles of 30 s at 95 °C (denaturing), 40 s at 56 °C (annealing), and 80 s at 72 °C (extending). This was followed by a final extending period of 2 min at 72 °C. The PCR product was stored in the refrigerator at 4 °C until used. Then, 4.5 μL of the product was mixed with 1.5 μL of a green loading dye to make 6 μL which was loaded into each well of a 1% agarose gel with 8 μL Midori dye using a multi-channel micro-pipette and 5 μL for size standard 100 bp ladder. This was analyzed by electrophoresis at 135 V for 30 min and transferred to the UV trans-illuminator where gel images were taken. The expected band size was 1000–1100 bp. Fifty-nine (59) samples were selected based on the quality of the amplicons. These successfully amplified PCR products were purified and sequenced on an Applied Biosystem Genetic Analyzer at Genoscreen, Lille, France.

#### 4.2.4. Sequence Analysis

The Cox1 and ITS2 sequences were assembled separately and edited with a 4.5 sequencer version: (Gene Codes Corporation; Ann Arbor, MI, USA). The sequences were aligned using BioEdit Version 7.0.9 and ClustalW software. The aligned sequences were compared with the sequences in the GenBank Nucleotide Database for species designation: (https://www.ncbi.nlm.nih.gov/nucleotide/ accessed on 30 March 2022). The nuclear ITS2 region between *S. haematobium* and *S. bovis* differs at five polymorphic sites, hence the sequence chromatograms were checked at these SNPs to identify any possible heterozygosity (Supplementary Figure S2). We constructed a Cox1 gene phylogenetic tree only using *S. bovis* sequences because the Cox1 *S. haematobium* gene is known to be poorly variable and a phylogenetic study revealed only two clusters in all the areas of repartition of the parasites [42]. The phylogenetic tree was constructed using MEGA version 6.0.6 (Pennsylvania State University, Philadelphia, PA, USA) using an HKY + G nucleotide substitution model identified as the best model describing data. The support for tree nodes was calculated with 1000 bootstrap iterations. The phylogenetic analysis includes *S. bovis* sequences from various African countries retrieved from GenBank databases with a minimum length of 778 bp (see Supplementary Table S1). The tree was rooted in the *S*. *haematobium* haplotypes of the present study. All sequences were uploaded onto the GenBank database (OL840258-OL840278).

#### 4.2.5. Microsatellite Genotyping

Microsatellite genotyping was performed on parasites from all sites except 5 and 6. A total of 1136 samples (Table 1) were individually genotyped with 18 microsatellite markers divided into two panels of 9 loci [43]. The multiplex PCR mix for each panel in two tubes was performed using the Qiagen^®^ multiplex PCR Kit (Qiagen, Hilden, Germany) according to the manufacturer’s standard amplification protocol. The forward primers were fluorescently labeled using 6-FAM, VIC, NED, and PET dyes (Applied Biosystems, Foster City, California, USA). The PCR mix consists of 5 μL Qiagen MM 2X, 1 μL of 10X microsatellite primer mix, and 4 μL DNA extract making a final volume of 10 μL. The thermal cycling was performed in a plate thermocycler, PerkinElmer 9600 Thermal Cycler (PerkinElmer, Waltham, MA, USA): pre-denaturing at 95 °C for 15 min, 40 cycles of 30 s at 94 °C (denaturing), 90 s at 56 °C (annealing), and 60 s at 72 °C (extending). This was followed by a final extending period of 30 s at 60 °C [43]. The microsatellite PCR products were sent to Genoscreen, Lille, France for genotyping. Each microsatellite locus was visibly peak called with GS500Liz size standard (Applied Biosystem) and GeneMarker software. Eighty percent of our samples were successfully amplified by 14 loci and were used for result analysis while 4 markers (C131, Sh4, Sh8, and Sh15), which amplified less than 20% of the samples, were excluded.

#### 4.2.6. Population Genetic Structure

Linkage disequilibria and departures from Hardy–Weinberg expectations were tested using exact tests (1200 permutations) adjusted for multiple tests using Bonferroni’s correction as implemented in the FSTAT software 2.9.3.2 [44]. We analyzed the genetic variability of schistosomes from each study site by computing the expected heterozygosity (He), number of alleles (A), allelic richness (Ar), and the inbreeding coefficient (Fis) in each microsatellite’s locus with FSTAT v.2.9.3.2 [44]. Heterozygosity (He) and allelic richness (Ar) between the populations were compared using the pairwise Friedman rank test followed by Nemenyi post hoc test.

Genetic structure was first assessed by calculating pairwise F_ST_ values between sites according to [45] using FSTAT version 2.9.3.2. A possible link between geographic (in Km) and genetic distances (Fst) was assessed using the Mantel test. Second, we used the principal components analysis (PCA) implemented in Genetix [46]. Because we sampled several miracidia per patient, these miracidia are related, which could influence the genetic structure. In order to assess a possible bias of our sampling strategy, we performed PCA by randomly sampling two miracidia per patient. Third, we used the Bayesian clustering approach implemented in the Structure software to determine the uppermost level of genetic structure [47]. We tested the number of clusters from K = 1 to K = 12, by computing three runs for each cluster which is made up of 10^6^ iterations after a “burn-in” period of 250,000 iterations with other parameters set by default and an admixture model. The mean logarithm probability for each cluster (K) was taken for the three runs with the *corrsieve* package in R. The ΔK-values were then computed in R to determine the probable cluster number from the total clusters (ΔK) tested according to Evanno et al. [48], from which we identified K = 2 as the most probable genetic clusters. Lastly, an additional 10 runs were computed for K = 2 using 10^6^ iterations and setting the same parameters as earlier described. The mean probability for a miracidium to belong to each cluster over the 10 runs was taken as Q-values, and we used Clumpp version 1.1.2 according to Francis R.M. [49], and Distruct version 1.1 according to Rosenberg N.A [50].

## 5. Conclusions

This study revealed that *S*. *haematobium*-*bovis* hybrids are predominant in *Schistosoma* eggs isolated in the urine samples of primary school-aged pupils in Nigeria. Our findings provide evidence that *S*. *haematobium* x *S*. *bovis* hybrids are common in Nigeria. Based on the high prevalence of *S*. *haematobium* x *S. bovis* hybrids, we advocate research priority on domestic and wild animals to investigate the role of zoonotic transmission.

## Figures and Tables

**Table 1:** Number of miracidia collected from participants and analyzed by Cox1 marker rapid diagnostic (RD) PCR to show the minimum percentage of *S. haematobium* x *S. bovis* hybrids.

Sampling Site	No. of Children Tested	No. Miracidia Genotyped	No. of Miracidia with Cox1 *S*. *bovis*	No. of Miracidia with Cox1 *S*. *haematobium*	Min. % of Hybrids (*S*. *bovis* Cox1)
1	10	90	90	0	100%
2	20	156	156	0	100%
3	18	152	28	124	18%
4	11	74	46	28	62%
5	3	30	30	0	100%
6	12	66	66	0	100%
7	12	84	84	0	100%
8	12	103	103	0	100%
9	17	164	164	0	100%
10	12	90	90	0	100%
11	20	193	193	0	100%
12	18	162	162	0	100%
Total	165	1364	1212	152	89%

**Table 2:** Prevalence of *S*. *haematobium*, *S*. *bovis* and *S*. *haematobium* x *S. bovis* hybrids for each sampling site based on Cox1 x ITS2 combinations of full genotyped 59 sub-samples.

Genotype		Site	1	2	3	4	5	6	7	8	9	10	11	12	Total Alleles (%)		
																*Sb* ITS2	*Sh* ITS2
COX1	ITS2																
*Sb*	*SbSb*		3	1	0	0	0	0	2	0	4	4	5	4	23 (39.0)	46 (39.0)	0 (0)
*Sb*	*ShSh*		2	0	4	2	0	0	0	0	0	1	0	0	9 (15.3)	0 (0)	18 (15.3)
*Sb*	*SbSh*		2	6	2	1	0	2	0	1	3	2	1	2	22 (37.2)	22 (18.6)	22 (18.6)
*Sh*	*SbSb*		0	0	0	0	0	0	0	0	0	0	0	0	0 (0)	0 (0)	0 (0)
*Sh*	*ShSh*		0	0	2	1	0	0	0	0	0	0	0	0	3 (5.1)	0 (0)	6 (5.1)
*Sh*	*SbSh*		0	0	2	0	0	0	0	0	0	0	0	0	2 (3.4)	2 (1.7)	2 (1.7)
Total			7	7	10	4	0	2	2	1	7	7	6	6	59 (100)	70 (59.3)	48 (40.7)

**Table 3:** Population genetic diversity indices per study and per locus. Mean and Standard error (SE) of expected heterozygosity (He), number of alleles detected (A), allelic richness (Ar), mean inbreeding coefficient (Fis).

Locus	Sh9	Sh3	C102	Sh1	Sh14	Sh6	C111	Sh7	Sh13	Sh11	Sh2	Sh5	Sh10	Sh12	Mean	SE
Site 1 n = 74																
He	0.633	0.824	0.000	0.704	0.875	0.365	0.608	0.560	0.696	0.454	0.877	0.841	0.536	0.335	0.593	0.247
A	5	10	1	6	11	5	5	4	8	5	10	9	7	4	6.429	2.848
Ar	4.590	9.603	1.000	5.550	10.934	4.947	4.645	3.636	7.330	4.980	9.574	8.710	6.014	3.956	6.105	2.774
Fis	0.569	0.241	NA	0.177	0.082	0.273	0.088	0.377	−0.026	0.416	0.411	0.415	0.501	0.048	0.275	0.196
Site 2 n = 206																
He	0.624	0.860	0.025	0.659	0.883	0.335	0.636	0.677	0.710	0.480	0.821	0.821	0.365	0.473	0.598	0.241
A	11	11	2	6	12	6	6	4	10	5	12	13	8	5	7.929	3.518
Ar	7.111	9.906	1.694	5.502	11.469	4.337	4.960	4.000	7.839	4.465	10.851	10.045	5.225	4.212	6.544	3.017
Fis	0.414	0.116	−0.010	0.013	0.063	0.090	0.039	0.468	−0.010	0.411	0.264	0.423	0.219	−0.086	0.172	0.191
Site 3 n = 219																
He	0.698	0.801	0.331	0.624	0.767	0.360	0.630	0.656	0.656	0.231	0.833	0.875	0.326	0.136	0.566	0.241
A	9	14	7	12	10	5	6	5	11	5	12	13	6	5	8.571	3.368
Ar	6.925	10.399	5.671	8.790	8.091	3.698	5.003	4.623	9.302	3.715	10.252	12.187	4.802	3.541	6.929	2.890
Fis	0.363	0.107	0.130	−0.006	0.151	0.344	0.108	0.094	0.103	0.562	0.335	0.202	0.399	0.158	0.218	0.157
Site 4 n = 76																
He	0.583	0.885	0.039	0.675	0.865	0.419	0.602	0.579	0.652	0.580	0.796	0.821	0.308	0.409	0.587	0.235
A	5	11	3	6	12	5	6	4	8	5	11	9	5	4	6.714	2.946
Ar	4.683	10.865	2.354	5.831	11.624	4.757	5.418	3.863	7.602	4.675	10.836	8.517	4.322	3.934	6.377	2.986
Fis	0.216	0.135	−0.009	−0.015	0.183	0.040	0.145	0.330	−0.062	0.655	0.392	0.513	0.333	−0.033	0.202	0.219
Site 7 n = 77																
He	0.585	0.781	0.208	0.569	0.731	0.000	0.659	0.534	0.822	0.194	0.750	0.758	0.666	0.703	0.569	0.253
A	8	9	2	5	7	1	6	3	11	2	7	7	7	4	5.643	2.925
Ar	6.624	8.526	2.000	4.714	6.506	1.000	5.133	2.998	9.906	2.000	5.969	6.680	6.897	4.000	5.211	2.591
Fis	0.399	0.179	0.126	0.214	0.088	NA	0.262	1.000	0.083	0.525	−0.167	0.330	0.675	0.196	0.301	0.298
Site8 n = 90																
He	0.732	0.802	0.163	0.525	0.673	0.000	0.629	0.509	0.736	0.229	0.711	0.671	0.677	0.664	0.552	0.245
A	9	8	2	5	7	1	4	4	5	4	7	8	9	7	5.714	2.525
Ar	8.112	7.737	2.000	4.448	6.387	1.000	3.859	3.669	4.999	3.424	6.643	7.285	7.628	6.179	5.241	2.242
Fis	0.700	0.213	−0.092	0.102	−0.008	NA	0.043	0.690	0.233	0.630	−0.102	0.109	0.636	0.089	0.249	0.300
Site 9 n = 87																
He	0.755	0.778	0.269	0.457	0.702	0.000	0.578	0.400	0.771	0.052	0.737	0.714	0.494	0.667	0.527	0.263
A	8	8	2	5	6	1	4	2	6	2	7	8	7	6	5.143	2.507
Ar	7.445	7.121	2.000	3.973	5.971	1.000	3.488	2.000	5.518	1.983	6.718	6.826	6.302	5.709	4.718	2.245
Fis	0.470	0.044	0.081	−0.080	−0.043	NA	0.376	0.826	0.408	−0.018	−0.192	0.159	0.349	0.104	0.191	0.275
Site 10 n = 77																
He	0.761	0.762	0.232	0.534	0.677	0.000	0.567	0.490	0.766	0.262	0.787	0.727	0.702	0.704	0.569	0.244
A	8	8	3	3	8	1	5	2	7	3	8	6	8	6	5.429	2.563
Ar	7.334	7.561	2.553	3.000	7.071	1.000	4.120	2.000	6.151	2.667	7.614	5.856	7.568	5.791	5.020	2.379
Fis	0.371	0.075	0.092	0.241	−0.113	NA	0.201	0.819	0.231	0.697	−0.059	0.089	0.719	0.105	0.267	0.298
Site 11 n = 61																
He	0.747	0.768	0.242	0.692	0.677	0.075	0.557	0.551	0.763	0.406	0.776	0.723	0.593	0.696	0.590	0.213
A	9	9	2	4	6	4	4	3	9	2	8	9	8	5	5.857	2.742
Ar	8.542	8.910	2.000	4.000	5.962	3.543	3.700	3.000	10.162	2.000	7.439	8.736	7.466	4.999	5.747	2.779
Fis	0.596	0.030	0.256	0.261	0.091	0.494	0.281	0.637	−0.063	0.707	−0.013	0.202	0.603	0.116	0.300	0.262
Site 12 n = 169																
He	0.760	0.752	0.200	0.619	0.725	0.045	0.608	0.420	0.816	0.123	0.738	0.712	0.468	0.728	0.551	0.259
A	10	11	4	6	7	2	5	4	11	3	8	9	8	7	6.786	2.914
Ar	7.487	8.692	3.555	4.973	6.448	1.895	4.220	3.188	9.908	2.340	6.648	7.411	6.542	4.797	5.579	2.401
Fis	0.513	0.167	0.038	0.134	−0.025	0.855	0.147	0.836	0.020	0.471	−0.034	0.174	0.665	−0.000	0.283	0.321
Total (n = 1136)																

**Table 4:** Pairwise genetic differentiation estimate (F_ST_—above the diagonal) and the Euclidian geographic distances (Km—below the diagonal) between the sampling sites. Most F_ST_ values are statistically significant (marked with an asterisk (*)) with the level of significance adjusted with Bonferroni correction (*p* < 0.0011). No link was observed between the geographical and genetic distances (Mantel test; *p* > 0.05).

Population Number	1	2	3	4	7	8	9	10	11	12
1	--	0.0104	0.0681 *	0.0441 *	0.1493 *	0.1454 *	0.1544 *	0.1295 *	0.1424 *	0.1579 *
2	5.6	--	0.0546 *	0.0187 *	0.1206 *	0.1286 *	0.1323 *	0.1157 *	0.1188 *	0.1348 *
3	72.7	68.2	--	0.0445 *	0.1195 *	0.1497 *	0.1387 *	0.1346 *	0.1179 *	0.1332 *
4	97.6	92.8	25.5	--	0.1274 *	0.1683 *	0.1688 *	0.1521 *	0.1181 *	0.1487 *
7	372.3	377.9	436.3	461.5	--	0.0358 *	0.0371 *	0.0318 *	0.0112	0.0209
8	311.7	317.3	376.4	401.6	24.3	--	0.0122	0.0052	0.0356 *	0.0241 *
9	298.9	304.5	363.3	388.5	36.1	13.2	--	0.0194	0.0485 *	0.0261 *
10	225.1	230.5	295.4	320.9	113.8	105.4	96.2	--	0.0244 *	0.0286 *
11	365.8	371.2	414.7	437.4	153.3	138.0	138.0	221.5	--	0.0220 *
12	497.9	501.3	506.5	518.5	439.3	419.8	415.4	471.3	291.0	--

**Table 5:** Number of miracidia collected from participants and genotyped with Cox1 and microsatellites for each of the sampling site.

Site No	Sampling Site	No. of Children	No. of Miracidia Collected	No. of Miracidia Genotyped with Cox1	No. of Miracidia Genotyped with Microsatellites
1	Ipogun	10	268	95	74
2	Ilara-Mokin	20	560	156	206
3	Alie Ilie	18	405	152	219
4	Lie Twon	11	279	74	76
5	Ikwo	3	82	30	0
6	Ohaozara	12	279	66	0
7	Onicha	12	277	84	77
8	Ishielu	12	278	103	90
9	Nkanu east	17	418	164	87
10	Anambra west	12	279	90	77
11	Gwer east	20	465	193	61
12	Jos north	18	417	162	169
	Total	165	4007	1364	1136

## Data Availability

Datasets generated for this report can be found on NCBI database for sequences and for microsatellite database (Supplementary Table S2).

## Supplementary Materials

The following supporting information can be downloaded at: https://www.mdpi.com/article/10.3390/pathogens11040425/s1, Table S1. Accession numbers for Supplementary Figure S1; Table S2. Microsatellite database; Figure S1. Maximum likelihood phylogenetic tree built with 21 (2 *S*. *haematobium* NigeriaHap1&2, and 19 *S*. *bovis* NigeriaHap3-21) haplotypes from the present study and haplotypes from Cameroon, Benin, Senegal, Cote d’lvoire, Kenya and Tanzania from Genbank database. See supplementary Table S1 for AN database; Figure S2. The sequence chromatograms show the pure and mixed signal in the nuclear ITS2 marker. The double sequence chromatogram (heterozygous) showing bi-parental inheritance of the nuclear DNA; Figure S3. Population genetic structure graph assessed by principal component analysis using 2 miracidia by patient. Each sampling site is represented by a dot. The first and second axis of the PCA represent 46.2% and 16.9% respectively of the total variation in allele frequency.
