# Supplementary material for: Population Genetic Structure and Hybridization of Schistosoma haematobium in Nigeria
Source: Pathogens. 2022 Mar 31;11(4):425. doi: 10.3390/pathogens11040425 (PMC9026724; doi:10.3390/pathogens11040425)
Supplement: Supplementary file 1 [file pathogens-11-00425-s001.zip › pathogens-1603065-supplementary/Supplementary Figure S1.pdf]

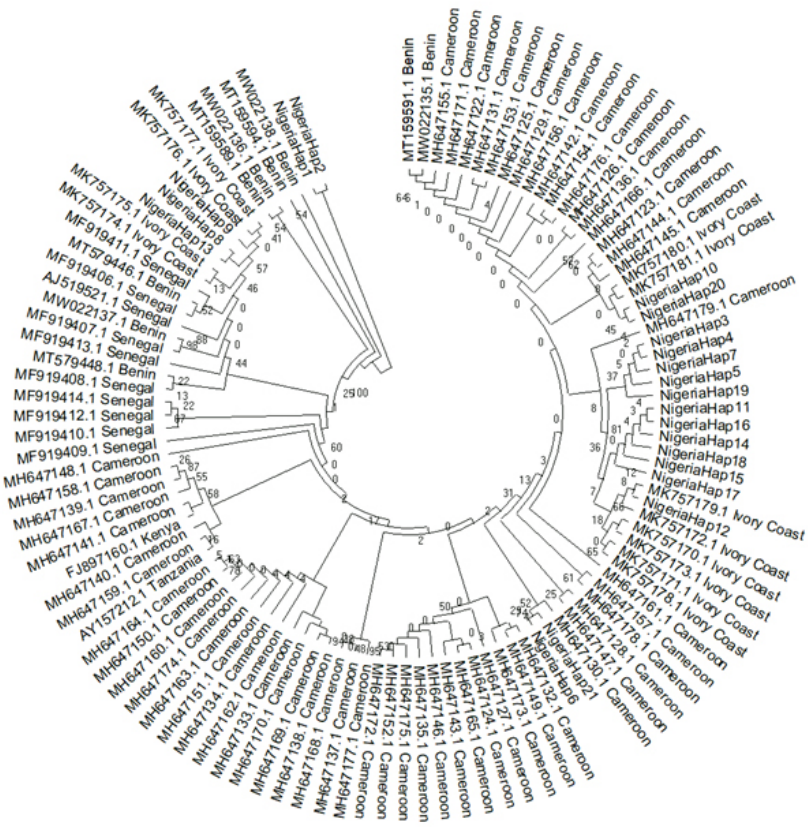

Figure S1. Maximum likelihood phylogenetic tree built with 21 (2 *S. haematobium* NigeriaHap1&2, and 19 *S. bovis* NigeriaHap3-21) haplotypes from the present study and haplotypes from Cameroon, Benin, Senegal, Cote d'lvoire, Kenya and Tanzania from Genbank database.
