# Supplementary material for: Population Genetic Structure and Hybridization of Schistosoma haematobium in Nigeria
Source: Pathogens. 2022 Mar 31;11(4):425. doi: 10.3390/pathogens11040425 (PMC9026724; doi:10.3390/pathogens11040425)
Supplement: Supplementary file 1 [file pathogens-11-00425-s001.zip › pathogens-1603065-supplementary/Supplementary Figure S2.pdf]

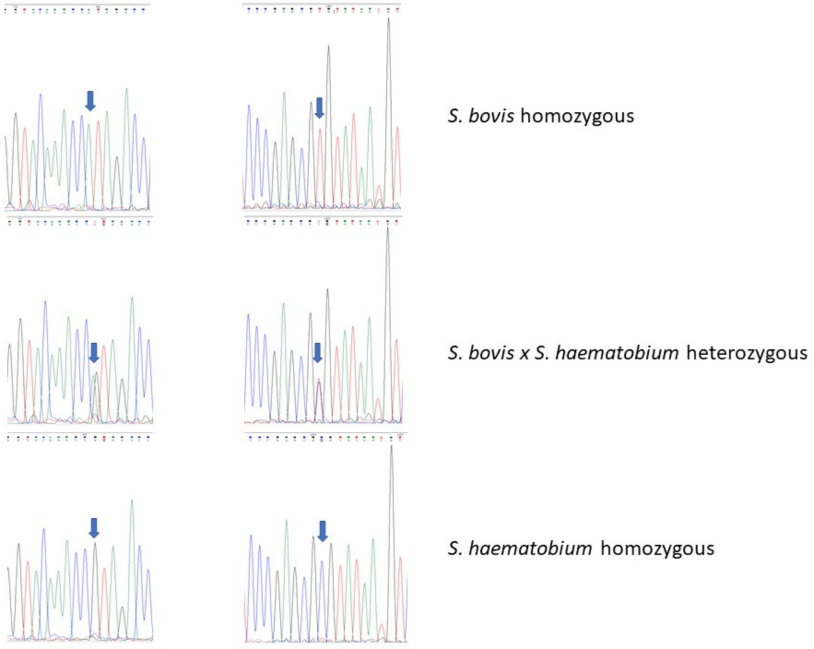

Figure S2. The sequence chromatograms show the pure and mixed signal in the nuclear ITS2 marker. The double sequence chromatogram (heterozygous) showing bi-parental inheritance of the nuclear DNA
