# Supplementary material for: Population Genetic Structure and Hybridization of Schistosoma haematobium in Nigeria
Source: Pathogens. 2022 Mar 31;11(4):425. doi: 10.3390/pathogens11040425 (PMC9026724; doi:10.3390/pathogens11040425)
Supplement: Supplementary file 1 [file pathogens-11-00425-s001.zip › pathogens-1603065-supplementary/Supplementary Figure S3.pdf]

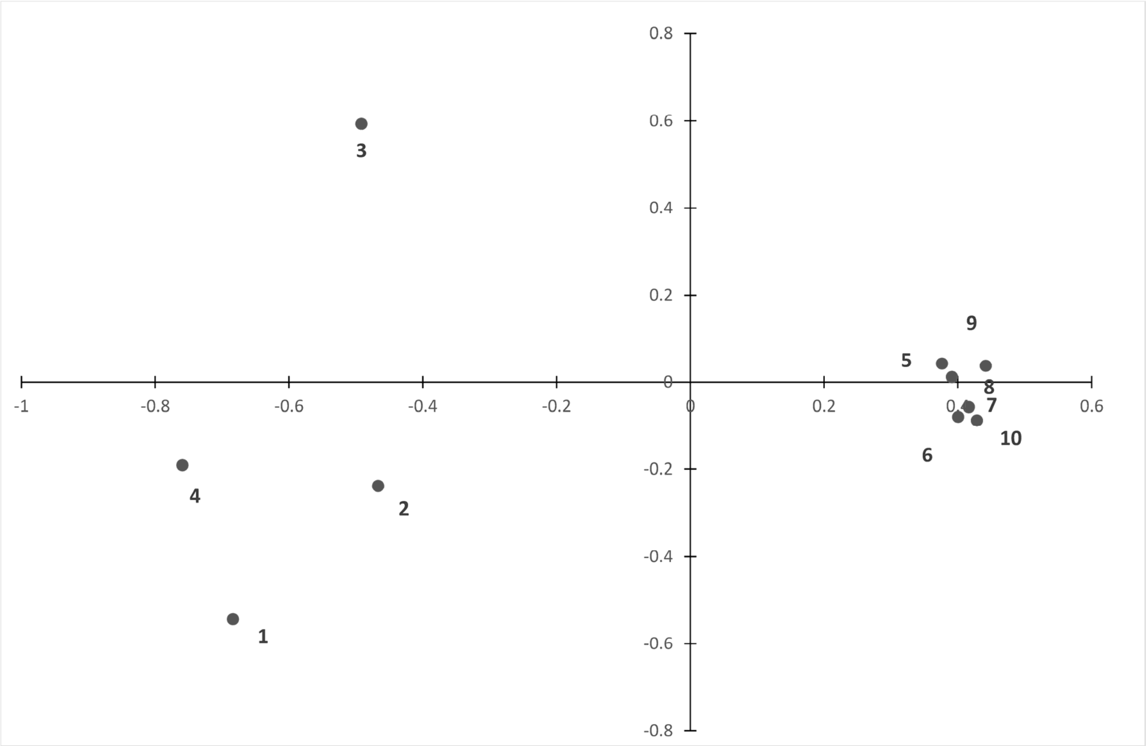

Figure S3. Population genetic structure graph assessed by principal component analysis using 2 miracidia by patient. Each sampling site is represented by a dot. The first and second axis of the PCA represent 46.2% and 16.9% respectively of the total variation in allele frequency.
